# Immune cells in liver injury: from pathogenic mechanisms to immunotherapy

**DOI:** 10.3389/fimmu.2026.1770949

**Published:** 2026-04-15

**Authors:** Hao Liao, Jie Zhang, JiHao Zhang, Yajin Chen, Xiong Chen, Yuan Meng, Jie Chen

**Affiliations:** 1 Department of Hepatobiliary Surgery, Sun Yat-Sen Memorial Hospital, Sun Yat-Sen University, Guangzhou, China; 2 Guangdong Provincial Key Laboratory of Malignant Tumor Epigenetics and Gene Regulation, Sun Yat-Sen Memorial Hospital, Sun Yat-Sen University, Guangzhou, China; 3 Hepatobiliary and Pancreatic Medical Treatment Center, People’s Hospital of Xinjiang Uygur Autonomous Region, Urumqi, China

**Keywords:** HSC, immunotherapy, immune cells, liver fibrosis, liver injury

## Abstract

Immune regulation is an essential process via which the immune system detects initial damage signals and initiates a response to preserve microenvironmental balance. In chronic liver illnesses (such as NAFLD, NASH, or viral hepatitis), a disruption in homeostasis results in sustained inflammation and the progression of liver fibrosis, a critical factor influencing long-term morbidity and death in patients. Liver inflammation is a multifaceted physiological reaction triggered by the combined influence of several signals from both internal and external sources. Recent advancements in single-cell and spatial transcriptomics have elucidated the mechanisms that regulate the heterogeneity, spatial distribution, and autophagic characteristics of diverse intrahepatic immune cell populations, such as macrophages, neutrophils, T cells, and non-classical lymphocytes. The immune responses meticulously govern the activation of hepatic stellate cells (HSCs), their subpopulation dynamics, and their transdifferentiation into myofibroblasts via a network of chemokines and cytokines. Due to the considerable unmet clinical requirements in NAFLD/NASH, thorough investigation of the mechanisms underlying liver inflammation and fibrosis has resulted in the identification of numerous promising treatment targets. This review intends to systematically elucidate the interactions between inflammatory mediators and immune cells, alongside the fibrotic signaling pathways and their regulation mechanisms in sick livers. It emphasizes recent clinical advancements in cell therapy for liver injury treatment, aiming to establish a theoretical basis for precise therapies in liver fibrosis.

## Introduction

Current studies have found that the main causative factors for liver injury are hepatic resection, drugs, alcohol, viral infections, cholestasis and autoimmunity (1). When these pathogenic factors cause liver injury, they will trigger the stress and inflammation of the liver’s innate and adaptive immune system in the first place (2). Inflammatory chemicals have the ability to stimulate hepatic stellate cells (HSCs), the main effector cells of liver fibrosis, leading to excessive buildup of extracellular matrix (ECM) and driving the progression of liver fibrosis (3). Thus, the crucial steps to initiate hepatocytes proliferation, suppress inflammation, improve hepatic lipid metabolism, and ultimately reverse liver fibrosis involve early activation and recruitment of reparative immune cells, transmission of signaling pathways, and inhibition of HSCs activation (4, 5).

At present, the options for treating liver injury are somewhat restricted, and liver transplantation is often the only viable approach to extend the patient’s lifespan (6). However, this method still encounters challenges such as a shortage of available liver donors or compatibility issues with the recipient (7). Immune regulation has emerged as a prominent area of research in the field of liver injury. It holds great promise in addressing the limitations of current therapeutic methods for liver injury. Hence, it is imperative to have a methodical compilation and analysis of the impact of immune regulation in liver injury.

## Molecular mechanisms of immune cells

In physiological equilibrium, a complex network of immune cells is spread throughout liver tissue, with these cells being broadly disseminated within the hepatic parenchyma and concentrated in the periportal areas (8). This spatially varied distribution is meticulously governed by hepatocyte homeostasis and paracrine signals from endothelial cells, while also being persistently modified by extrahepatic influences, including microbial metabolites sent through the gut-liver axis (9). In this immunological environment, each cell population has distinct physiologic impacts (10). Liver-resident macrophages (Kupffer cells) establish a robust phagocytic network through the expression of the immunoglobulin superfamily complement receptor (CRIg/VSIG4), which facilitates the clearance of pathogens, including Gram-positive bacteria, from the bloodstream (11). In non-inflammatory situations, these macrophages cooperate with dendritic cells to execute antigen-presenting tasks, preserving the liver’s immunological tolerance via specialized immune checkpoint signaling (12). The lymphocyte population in the liver comprises conventional and non-conventional T cells, B cells and NK cells collectively forming the liver’s immunological surveillance defense (13). When the liver sustains damage (e.g., from NASH, NAFLD, or parasite infection), chemokines and cytokines generated by injured cells swiftly initiate immune mobilization (14). Neutrophils and monocyte-derived macrophages are the initial responders to the injury site, where they eliminate cellular debris via phagocytosis (5). T cells, B cells, and NK cells subsequently stimulate stromal cells and resident macrophages through the secretion of pro-inflammatory mediators (5). This multicellular signaling cascade ultimately intensifies inflammatory infiltration in the liver and propels the pathological progression from parenchymal damage to fibrosis.

## Macrophages

Hepatic macrophages, the predominant immune cell population in the liver, demonstrate significant phenotypic variety and functional adaptability (15). This population is predominantly composed of resident Kupffer cells (KCs) and a limited number of monocytes-derived macrophages (MoMFs) (15). KCs function as “immune sentinels” in blood arteries, exhibiting heightened sensitivity to stress and damage signals from both intrahepatic and extrahepatic settings (16). At the outset of inflammation, they swiftly increase the macrophage population by enlisting substantial numbers of bone marrow-derived monocytes into the liver (17, 18). These cells are pivotal in liver damage, inflammation, and fibrosis, with their functions adapting in response to alterations in environmental cues.

Recently, a study demonstrated that adipocyte death triggers the activation and intrahepatic infiltration of C-C motif chemokine receptor 2 (CCR2^+^) macrophages. This process activates the lipolysis, resulting in the release of free fatty acids (FFAs) from the liver, which leads to steatohepatitis (19). Furthermore, Du et al. found that T-cell immunoglobulin and mucin-domain-containing protein-3 (TIM-3^+^) macrophages mitigate methionine and choline-deficient diet (MCD)-induced steatohepatitis by suppressing reactive oxygen species (ROS) generation and subsequent ROS-mediated secretion of IL-1β and interleukin-18 (IL-18) (20).

The process of macrophages phagocytosis of apoptotic cells plays a crucial role in the regenerative regressive response, which aids in the repair of liver-injured tissue by successfully preserving tissue homeostasis and minimizing excessive inflammation (21, 22). A study found that alcohol enhances the process of KCs phagocytosis by causing the movement of mixed lineage kinase domain‐like pseudokinase (MLKL) from macrophages to phagosomes and lysosomes. This helps prevent damage to hepatocytes and reduces inflammation in alcohol-related liver disease (ALD) (23). Moreover, increased expression of T-cell immunoglobulin and mucin structural domain-containing protein-4 (TIM-4^+^) macrophages also enhance the phagocytosis of KCs and speeds up the regeneration of injured hepatocytes by stimulating the production of IL-10 and reducing the expression of TNF-α, thereby counteracting inflammation caused by liver ischemia-reperfusion injury (IRI) (24). Additionally, Chen et al. demonstrated that A+T rich interaction domain protein 3a (Arid3a) exacerbates cholestatic liver injury by hindering mertk-dependent macrophages phagocytosis of apoptotic cholangiocytes during cholestasis (25).

**Figure 1:**
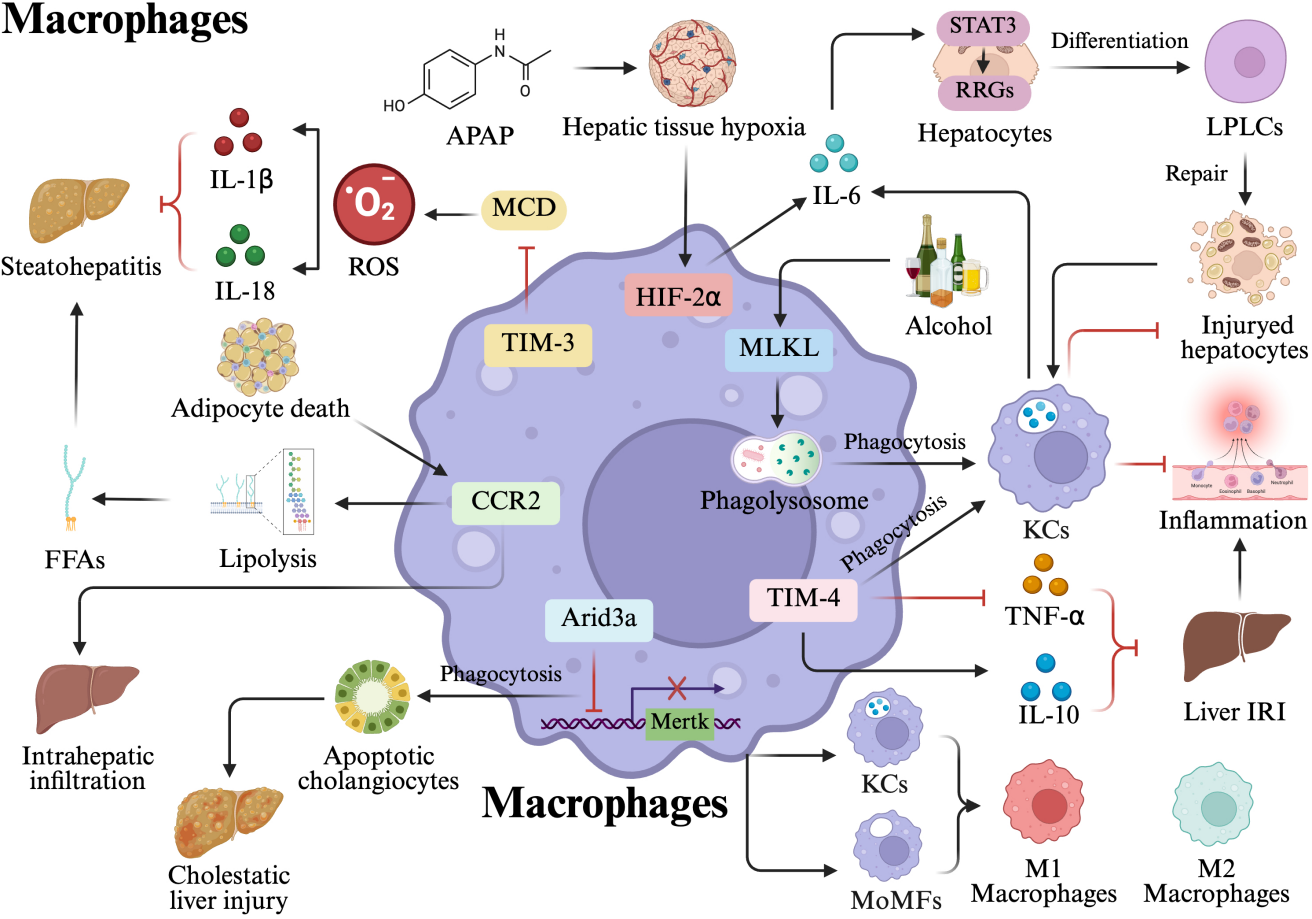
Mechanisms of macrophage-mediated liver injury. Pro-inflammatory and injury mechanisms: Adipocyte death triggers the infiltration of CCR2^+^ macrophages, which activate lipolysis and the release of FFAs, leading to steatohepatitis. During cholestasis, Arid3a exacerbates injury by inhibiting mertk-dependent phagocytosis of apoptotic cholangiocytes. Anti-inflammatory and Protective Signaling: TIM-3^+^ macrophages mitigate MCD-induced steatohepatitis by suppressing ROS generation and the subsequent secretion of IL-1β and IL-18. In ALD, alcohol-induced MLKL translocation to phagosomes/lysosomes enhances KCs phagocytosis, reducing inflammation. Phagocytosis and Homeostasis: Phagocytosis of apoptotic cells by macrophages is essential for the regenerative regressive response and preserving tissue homeostasis. Similarly, TIM-4^+^ macrophages counteract IRI by enhancing phagocytosis and stimulating IL-10 production while reducing TNF-α. Hepatocyte Regeneration and Reprogramming: Following APAP-induced injury or periportal damage, hypoxia upregulates HIF-2α in macrophages, activating the IL-6-STAT3 axis. This signaling pathway promotes hepatocyte multiplication and the dedifferentiation of hepatocytes into LPLCs via the reactivation of RRGs to facilitate tissue repair. This figure was created using BioRender (https://biorender.com/).

Interleukin-6 (IL-6) has a crucial role in stimulating the regrowth of the liver and enhancing the multiplication of hepatocytes through the IL-6-signal transducer and activator of transcription 3 (STAT3) (IL-6-STAT3) axis (26, 27). A study found that acetaminophen (APAP)-induced hepatic injury and hypoxia, which in turn upregulates the expression of hypoxia inducible factor-2α (HIF-2α) in hepatic macrophages, and subsequently HIF-2α reprograms macrophages to produce IL-6 to activate STAT3 in hepatocytes, which protects hepatocytes from APAP-induced liver injury (28). Furthermore, Li et al. found that following periportal liver injury, activated KCs in the affected region have the ability to reactivate reprogramming/progenitor-related genes (RRGs) via the IL-6-STAT3 axis. This process facilitates the dedifferentiation of hepatocytes into liver progenitor-like cells (LPLCs) and aids in the repair of the injured hepatocytes (**Figure 1****) (**29).

## Dendritic cells

Alongside the significant adaptability demonstrated by macrophages, the variety of the liver’s immunological environment is further illustrated by the highly specialized functional population of dendritic cells (DCs) (30). Macrophages directly influence the progression and resolution of inflammation via phenotypic flipping, whereas dendritic cells function as the “conductors” of the immune response (30). By detecting pathogen-associated molecular patterns (PAMPs) or damage-associated molecular patterns (DAMPs) in the microenvironment, they collaborate with macrophages to regulate pro-inflammatory or immunosuppressive signals in the liver (31).

DCs are crucial antigen-presenting cells in the immune system, categorized into two primary subtypes: plasmacytoid dendritic cells (pDCs) and conventional dendritic cells (cDCs) (32). Mature dendritic cells expand in fibrotic livers, however their precise function in the progression of liver fibrosis is still ambiguous (33). pDCs have elevated numbers of endosomal nucleic acid-sensing toll-like receptor 7 (TLR7) and toll-like receptor 9 (TLR9). They also release significant quantities of type I interferon (IFN-I), which has both inflammatory and immunosuppressive properties (34). cDCs are a type of specialized cells that convey antigens. They can be split into two subgroups: type I classical dendritic cells (cDC1s) and type II classical dendritic cells (cDC2s) (35). pDCs and cDCs recognize dynamic changes in the hepatic microenvironment at the early stage of liver injury, thereby modulating the inflammation and fibrosis of liver tissue in the first instance (36).

Notably, a study found that C-C motif chemokine receptor 9 (CCR9)-deficient pDCs exhibit enhanced migration to the liver during concanavalin A (ConA)-induced inflammation and exert immunosuppressive effects, thereby reducing acute liver inflammation (37). Furthermore, pDCs stimulated the production of interleukin-35 (IL-35) by regulatory T cells (Tregs), thereby reducing the severity of acute liver injury (ALI) induced by ConA (38).

**Figure 2:**
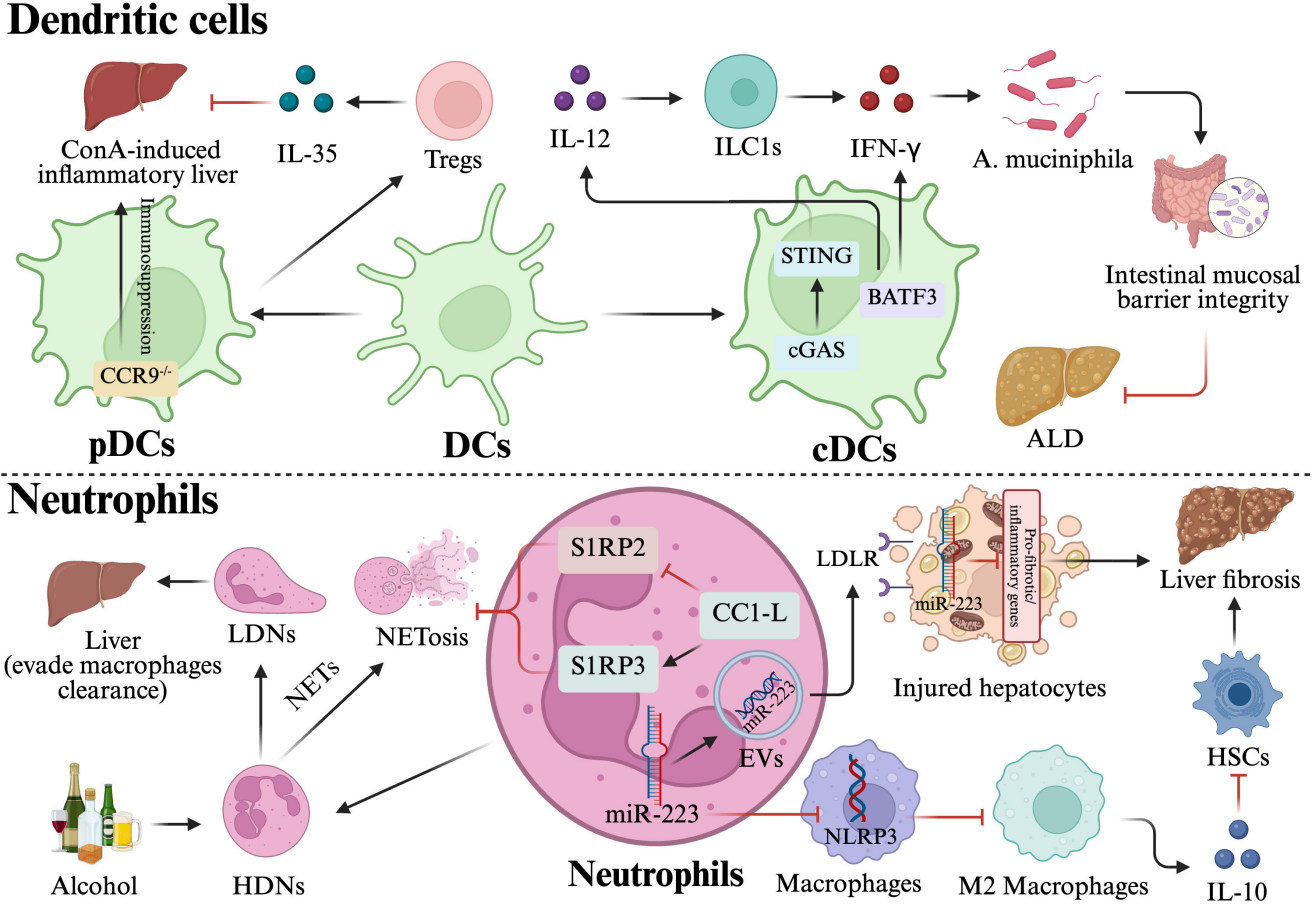
Dual regulatory roles of dendritic cells and neutrophils in liver injury and fibrosis. DCs: pDCs: These cells sense nucleic acids via TLR7/9 and release IFN-I to balance inflammation and immunosuppression. In ConA-induced injury, CCR9-deficient pDCs migrate to the liver to exert immunosuppressive effects, while pDC-stimulated IL-35 production by Tregs further mitigates ALI. cDCs: Resident cDC1s respond to DAMPs via the cGAS-STING-IL-12 axis, activating a protective cDC1-ILC1 circuit that produces IFN-γ to shield hepatocytes from apoptosis. Furthermore, cDC1s maintain intestinal barrier integrity and *A. muciniphila* abundance, thereby reducing the incidence of ALD. Neutrophils: NETosis and Tissue Injury: The release of NETs and subsequent NETosis sustain inflammation and drive host tissue damage through DAMP release. In ALD, alcohol-stimulated HDNs trigger NETosis before transforming into LDNs to evade clearance. MiR-223 Delivery: Neutrophil-derived miR-223 acts as a critical molecular rheostat. It suppresses NLRP3 in macrophages to promote M2 polarization and IL-10 production, thereby inhibiting HSCs activation and fibrosis. Inter-cellular communication: In lipotoxic environments, neutrophils function as “molecular delivery vehicles,” transferring miR-223-enriched EVs to hepatocytes via the LDLR-APOE pathway. This targeted delivery inhibits inflammatory and fibrotic gene expression, preserving hepatic metabolic equilibrium. Protective receptors: Neutrophils highly expressing CC1-L regulate the S1RP3/S1RP2 balance to effectively prevent liver injury caused by NETosis. This figure was created using BioRender (https://biorender.com/).

In response to liver injury, the release of DAMPs initiates a cGAS-STING-dependent IL-12 response in resident cDC1s, which activates a protective cDC1-ILC1 circuit that generates interferon-γ (IFN-γ) to protect hepatocytes from apoptosis and mitigate systemic inflammation, thus preserving the integrity of intestinal tight junction proteins and maintaining both hepatic and gut barriers (39).Moreover, the abundance of *Akkermansia muciniphila* (*A. muciniphila*) in the intestinal tract is maintained by conventional type 1 DCs (cDC1s) that relies on the basic leucine zipper transcription factor ATF-like 3 (BATF3). This is achieved by the secretion of IL-12 and IFN-γ, which help preserve the integrity of intestinal mucosal barrier and decrease the occurrence of ALD (**Figure 2**) (40).

## Neutrophils

Although DCs typically capture and present antigens to generate specific immune responses, the liver’s innate defense also depends on the swift mobilization of specialized phagocytes (41). Neutrophils are the primary cellular defense that enhances this intricate antigen-processing machinery in reaction to liver injury (42).

Neutrophils serve as the primary defense sentinels against bacterial infection and can be quickly activated in response to intestinal dysbiosis and heightened intestinal permeability (42). They combat infection by engulfing bacteria through phagocytosis and/or by releasing neutrophil extracellular traps (NETs) (43). Activated chemokines and liver sinusoidal endothelial cells (LSECs) facilitate the recruitment of neutrophils after liver injury (41). Moreover, the intrahepatic infiltration of neutrophils can assist in the process of healing by producing hepatocyte growth factor (HGF) and facilitating the transformation of macrophages into a reparative phenotype (44).

The release of NETs is accompanied by neutrophils death (NETosis), the formation of which sustains inflammation and leads to host tissue damage through the release of DAMPs (45, 46). Cho et al. found that alcohol immediately stimulates high-density neutrophils (HDNs) to release NETs, which form NETosis and lead to liver tissue damage, followed by the transformation of HDNs into low-density neutrophils (LDNs) that remain in the liver to evade macrophages clearance (47). Furthermore, a study found that neutrophils with a high expression of the carcinoembryonic antigen–related cell adhesion molecule 1-long (CC1-L) play a positive role in regulating sphingosine-1-phosphate receptor 3 (S1RP3) and a negative one in regulating sphingosine-1-phosphate receptor 2 (S1RP2). As a result, they effectively prevent liver injury caused by NETosis (48).

It is widely believed that microRNA-223 (miR-223) is the predominant microRNA found in neutrophils (49). A study indicated that neutrophils-derived miR-223 functions as a suppressor of NLR family pyrin domain containing 3 (NLRP3) in macrophages, so promoting macrophages M2 polarization and enhancing the production of IL-10. Consequently, it hinders the activation of HSCs and mitigates liver fibrosis (50). Moreover, in a lipotoxic environment, hepatocytes selectively internalize miR-223-enriched extracellular vesicles (EVs) originating from neutrophils through the low-density lipoprotein receptor (LDLR)- apolipoprotein E (APOE) recognition pathway, therefore converting neutrophils from simple pro-inflammatory agents into “molecular delivery vehicles.” Via miR-223, they directly inhibit the production of inflammatory factors and fibrosis-associated genes in hepatocytes, so effectively preventing the progression from basic steatosis to advanced hepatitis and fibrosis, while preserving the liver’s metabolic equilibrium (**Figure 2**) (51).

## NK cells

While neutrophils are essential in the immediate response to liver injury and initial repair, the liver’s innate immune system functions through multiple cell populations. Alongside these swiftly reacting phagocytes, the liver is also abundant in another crucial effector cell-natural killer (NK) cells. NK cells are integral to antiviral and anticancer immunity, as well as essential in modulating the activation of hepatic stellate cells and preserving hepatic immunological homeostasis (52).

**Figure 3:**
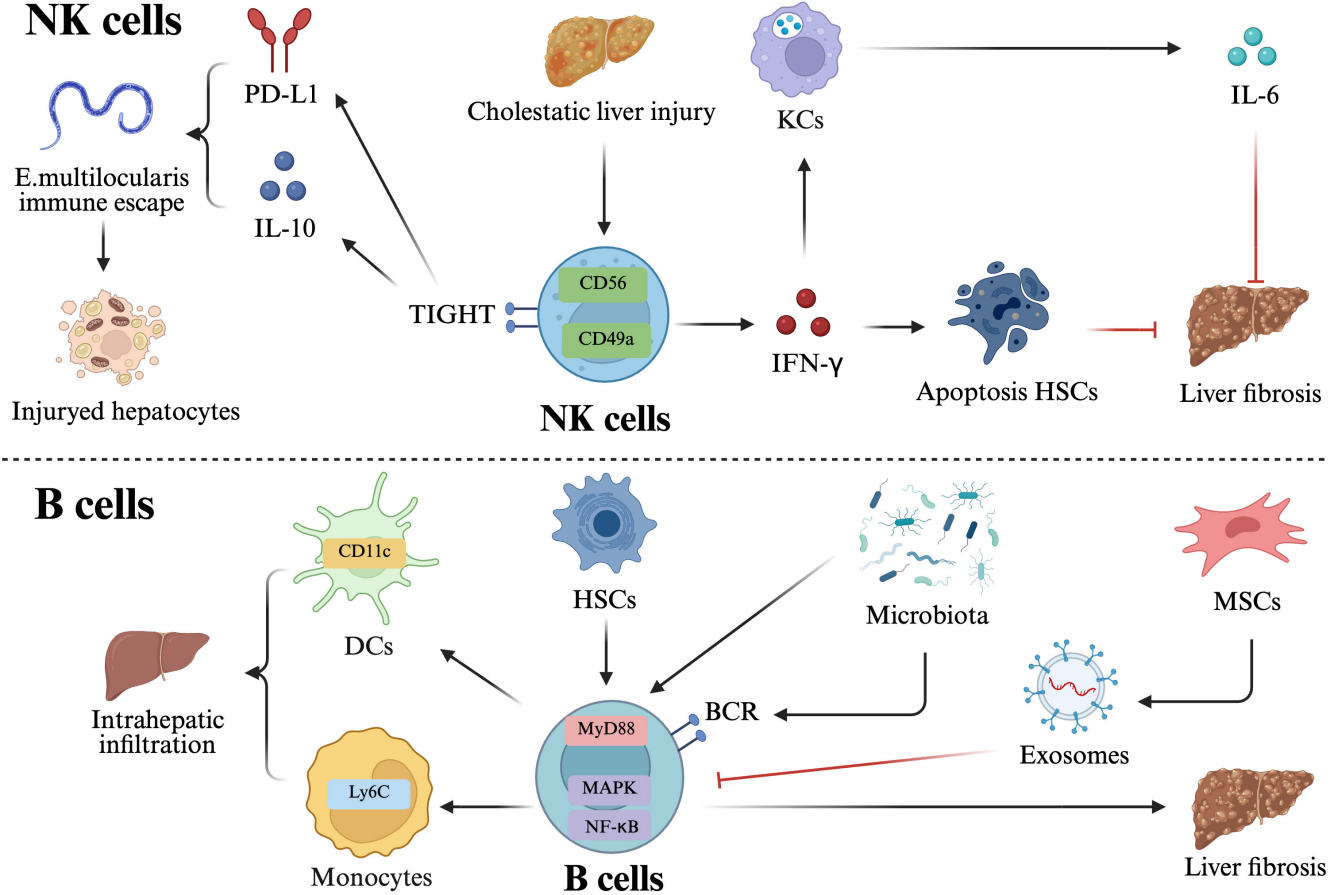
Multifaceted roles of NK cells and B cells in liver Injury and fibrosis. NK cells: NK cells: NK cells function as a critical antifibrotic subset by selectively identifying and eliminating activated HSCs. The release of IFN-γ by NK cells induces HSCs death and inhibits collagen synthesis, directly decelerating the progression of fibrosis. In the context of biliary obstruction, activated NK cells stimulate KCs to produce IFN-γ-dependent IL-6, which serves to mitigate cholestatic liver injury. Pro-inflammatory and immunosuppressive NK cell subsets: In cases of Echinococcus multilocularis infection or AE, regulatory NK cell subsets (CD56^bright^TIGIT^+^ in humans and CD49a^+^TIGIT^+^ in mice) are significantly elevated. These cells co-express PD-L1 and secrete IL-10, creating an inhibitory milieu that facilitates larval proliferation and leads to chronic liver injury. B cells: Resident B cells, activated by HSCs-induced MyD88 signaling, promote liver fibrosis by increasing the infiltration of migratory CD11c^+^ dendritic cells and Ly6C^+^ monocytes. In NASH, B-cells activation involves a synergistic interplay between innate (MyD88) and adaptive (BCR) signaling systems. Therapeutic modulation of B-cell activity: MSCs can counteract B-cell-mediated liver fibrosis. This therapeutic effect is achieved via exosomal delivery, which selectively inhibits the MAPK and NF-κB signaling pathways associated with B-cells activation. This figure was created using BioRender (https://biorender.com/).

NK cells are an essential element of the innate immune system and derive from CD34^+^ hematopoietic stem cells (53). As an antifibrotic cell subset, NK cells can selectively identify and eliminate active HSCs (54). Moreover, the IFN-γ they release triggers death in HSCs and suppresses collagen synthesis, so directly decelerating the advancement of fibrosis (55). During the initial phase of cholestatic damage, Biliary obstruction activates NK cells, which stimulate KCs to produce IFN-γ-dependent IL-6, thereby inhibiting cholestatic liver injury. (56). A separate study revealed that patients with Alveolar echinococcosis (AE) and mice livers infected with *Echinococcus multilocularis* (*E. multilocularis*) exhibited elevated quantities of regulatory CD56^bright^ NK cells expressing high levels of TIGIT and mouse CD49a^+^ NK cells, respectively. These cells co-express elevated levels of PD-L1 and produce increased amounts of IL-10, thereby fostering an inhibitory milieu that permits Echinococcus larvae to proliferate uncontested in the liver, resulting in chronic liver injury (**Figure 3****) (**57).

## T cells and B cells

Following the elucidation of the innate immune defense comprising macrophages, neutrophils, dendritic cells, and NK cells, the emphasis of the immune response transitions from a general, nonspecific initial defense to the adaptive immunological phase facilitated by T and B cells. In contrast to innate immunity cells that identify universal molecular characteristics of pathogens via pattern recognition receptors (PRRs), the adaptive immune system attains accurate recognition and response to individual antigenic epitopes through its highly varied surface antigen receptors (58). This transition signifies a fundamental change in the body’s defense mechanisms from “collective resistance” to “individual elimination,” while also granting the immune system the capacity to form immunological memory via clonal proliferation and differentiation (59). In this meticulously organized defense system, T cells, central to cellular immunity, direct immune polarization by either directly eliminating target cells or releasing cytokines, whereas B cells, upon antigen stimulation and T-cell support, differentiate into plasma cells and perform humoral immune functions by producing high-affinity antibodies (60). Collectively, they form the most advanced and resilient defense against pathogen intrusion.

Despite B cells representing a minor fraction of immune cells in the liver, their infiltration with chronic liver injury is nevertheless evident (61). A study revealed that HSC-induced activation of the MyD88 signaling pathway in resident B cells increases the hepatic infiltration of migratory CD11c^+^ dendritic cells and Ly6C^+^ monocytes, hence facilitating liver fibrosis (62). Moreover, B-cell activation in NASH encompasses both MyD88 signaling and B-cell receptor (BCR) signaling, indicating a synergistic interplay between innate and adaptive antigen recognition systems (63). A separate study revealed that mesenchymal stem cells (MSCs) can impede B-cell-mediated liver fibrosis by utilizing exosomes to selectively target and inhibit the MAPK and NF-κB signaling pathways associated with B-cell activation in the liver (**Figure 3****) (**64).

T cells, such as CD4^+^ T cells and CD8^+^ T cells, are the primary biological constituents of the adaptive immune system (65). CD8^+^ T cells serve as the principal effector cells of the spontaneous immune response in the liver, identifying antigen-peptide complexes on hepatocyte surfaces through MHC-I molecules, then releasing perforin and granzymes, or directly triggering apoptosis in target hepatocytes via the FasL-Fas pathway (66). CD4^+^ T cells meticulously modulate the hepatic immune microenvironment by differentiating into subpopulations exhibiting considerable functional diversity: Th1 cells activate KCs through the secretion of IFN-γ and TNF-α, thereby amplifying the pro-inflammatory response linked to acute liver injury; Th2 cells, while capable of counteracting the Th1 response, produce IL-13, a potent pro-fibrotic factor that directly stimulates HSCs; Th17 cells, conversely, recruit substantial numbers of neutrophils to the liver via IL-17 secretion, significantly intensifying inflammatory cascades and parenchymal damage in pathological conditions such as ALD and NASH (67).

Tissue-resident memory T (TRM) cells are a type of memory CD8^+^ T cells that remain in tissues without circulating (68). A study found that the capicua (CIC)-ETS translocation variant 5 (ETV5) axis suppresses the formation of CD8^+^ TRM cells, hence shielding the liver from damage caused by CD8^+^ TRM cells (69). Furthermore, Li et al. found that Itaconic exerts its inhibitory effect on runt-related transcription factor 3 (RUNX3) expression by impeding the DNA demethylation process of RUNX3 in CD8^+^ TRM cells. Consequently, it hinders the intrahepatic infiltration of CD103^+^ TRM cells dependent on RUNX3 in primary sclerosing cholangitis (PSC) and mitigates its enduring cytotoxic impact on the PSC liver (70). Additionally, CD8^+^ TRM cells attract HSCs by a mechanism that relies on C-C motif chemokine receptor 5 (CCR5) during the resolution of NASH. This process increases the likelihood of activated HSCs undergoing apoptosis through FasL-Fas signaling, hence facilitating the regression of liver fibrosis (71).

Regulatory cells (Tregs) are regarded as a crucial element of the adaptive response to tissue damage and are vital for efficient tissue repair and recovery (72). They can impede liver fibrosis through the production of the anti-inflammatory cytokine IL-10 (73). Research indicates that CCL2, synthesized by ILC1, attracts and activates Tregs, which subsequently promote macrophage polarization to the M2 phenotype by the secretion of IL-10, thus mitigating liver injury by equilibrating the inflammatory milieu (74). Additionally, TLR4 on Tregs enhances IL-10 synthesis through the MyD88/ERK/CREB signaling cascade, therefore mitigating injury induced by hepatic ischemia-reperfusion (75).

**Figure 4:**
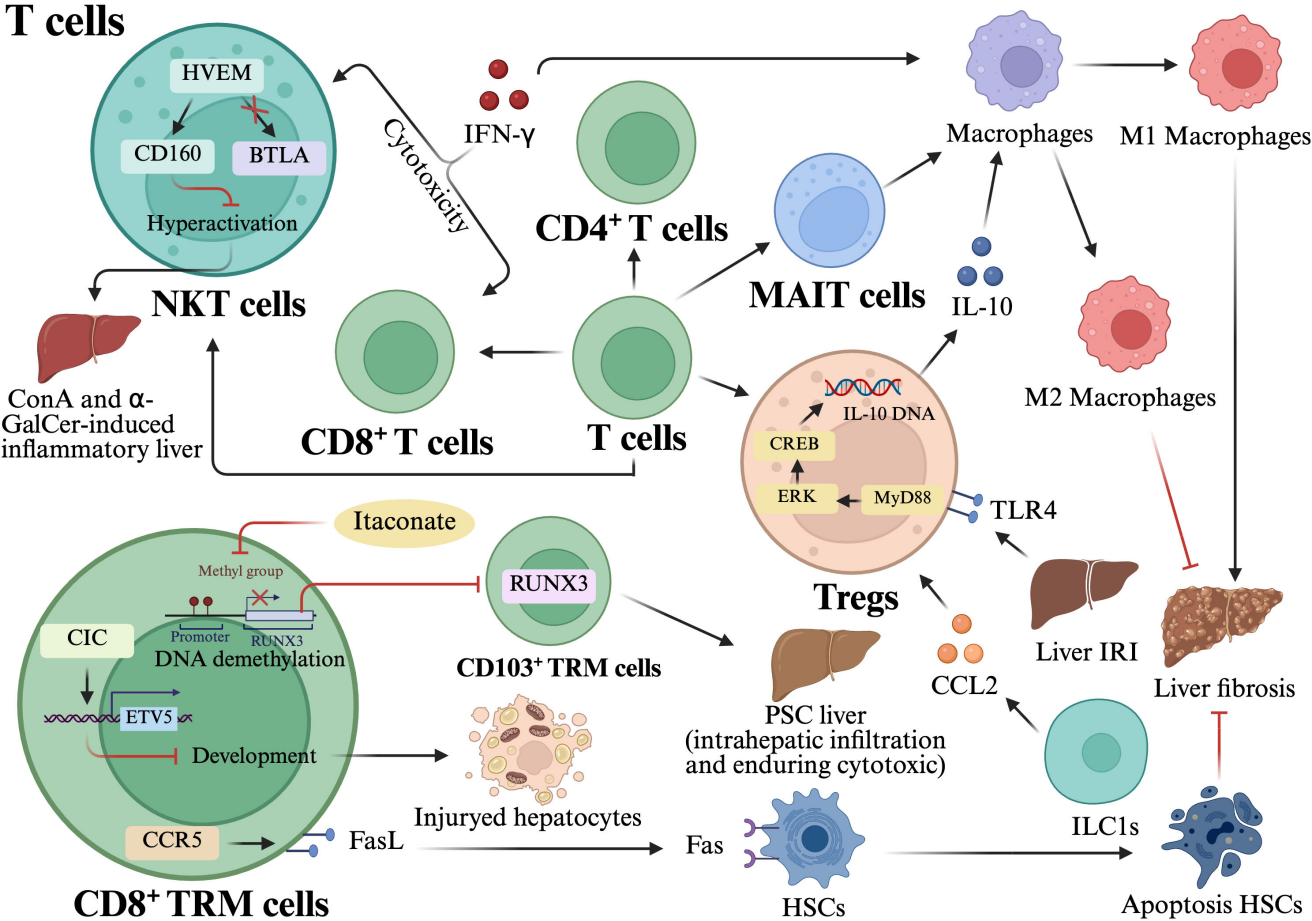
Orchestration of liver injury and repair by conventional and non-conventional T cell subsets. CD8^+^ TRM cells: Pro-inflammatory and cytotoxic roles: The CIC-ETV5 axis acts as a molecular shield by suppressing TRM cells formation. Itaconate further limits liver damage in PSC by inhibiting RUNX3-dependent DNA demethylation, thereby preventing the intrahepatic infiltration of cytotoxic CD103^+^ TRM cells. Pro-resolving roles: During NASH resolution, CD8^+^ TRM cells recruit HSCs via a CCR5-dependent mechanism. This interaction facilitates the regression of fibrosis by promoting HSCs apoptosis through FasL-Fas signaling. Tregs: Anti-inflammatory signaling: ILC1-derived CCL2 recruits and activates Tregs, which subsequently promote M2 macrophage polarization via IL-10 secretion to balance the inflammatory milieu. Signaling cascades: In hepatic ischemia-reperfusion injury, TLR4 expression on Tregs enhances IL-10 synthesis through the MyD88/ERK/CREB signaling pathway to mitigate tissue damage. NKT cells: In acute hepatitis, the CD160-HVEM axis delivers negative regulatory signals to NKT cells to prevent hyperactivation-induced injury. Conversely, IFN-γ can enhance NKT cell cytotoxicity and polarize macrophages toward a pro-fibrotic M1 phenotype. MAIT cells: Signal-dependent outcomes: TCR-mediated activation triggers the release of anti-fibrotic molecules, while cytokine-mediated activation promotes pro-inflammatory and fibrotic cytokine release. Macrophage modulation: MAIT cells drive M1 pro-fibrotic macrophage transformation; conversely, blocking MAIT cell activation promotes a shift toward M2 reparative morphologies, facilitating the reversal of fibrosis. This figure was created using BioRender (https://biorender.com/).

Alongside conventional T cells, non-conventional T cell subsets, including natural killer T (NKT) cells and mucosa-associated invariant T (MAIT) cells, significantly contribute to liver injury and have a dual role in liver fibrosis. NKT cells are a form of innate T cells that possess characteristics common to both NK cells and T cells. They may be classified into two primary subtypes: type I NKT cells and type II NKT cells (76). The severity of liver injury is often dictated by the balance between NKT cell subsets; Type I NKT cells promote inflammatory progression, while Type II NKT cells provide a protective effect by neutralizing Type I-driven inflammatory signals and reducing subsequent liver injury (77). Kim et al. found that in cases of acute hepatitis induced by α-galactose ceramide (α-GalCer) and ConA, the CD160-herpes virus entry mediator (HVEM) axis transmits B and T lymphocyte associated (BTLA)-independent negative regulatory signals to NKT cells and inhibits liver injury induced by NKT cells hyperactivation (78). Moreover, a study demonstrated that IFN-γ increased the cytotoxicity of NKT cells and CD8^+^ T cells in PSC liver by modifying their characteristics. Additionally, it also increased the ratio of macrophages M1/M2 polarization in the liver, which contributed to the advancement of liver fibrosis (79). Mucosa-associated invariant T (MAIT) cells are unique T cell subsets that can be activated by their T-cell antigen receptor (TCR) and/or cytokines receptors. Their activation pattern affects hepatic fibrogenesis (80). TCR activation signals MAIT cells to release anti-fibrotic effector molecules, while cytokines activation releases pro-inflammatory and fibrotic cytokines (81). A study indicated that MAIT cells stimulate the transformation of macrophages into an M1 pro-fibrotic phenotype. Blocking the activation of MAIT cells stops the advancement of fibrosis and encourages the reversal of fibrosis by altering the balance between pro-fibrotic and reparative macrophage profiles, leading to a higher occurrence of M2 reparative morphologies(**Figure 4****) (**82).

While we have initially clarified the geographic distribution and phenotypic evolution of macrophages, neutrophils, and other lymphocyte types in liver injury (83), current research encounters two significant challenges: The functionality of immune cells is significantly influenced by intricate microenvironments, such as gut-liver axis communication, and research concentrating on a singular cell type frequently neglects to encompass the comprehensive interactions within the immune network (84, 85). Secondly, the majority of study findings are based on animal models, and disparities in immune system development between humans and mice restrict the applicability of mechanistic studies to clinical practice (86). In the future, we should employ single-cell spatial proteomics and organoid-on-a-chip technologies to further corroborate these immune regulatory axes in human liver specimens (87). The emphasis should transition to investigating methods for accurately prompting immune cells to transition from a pro-inflammatory/pro-fibrotic phenotype to a repair/pro-regenerative phenotype, rather than merely eliminating cells, thereby attaining meticulous regulation of liver homeostasis.

## Clinical applications of immunotherapy

Liver regeneration is inhibited during liver injury, and excessive fibrosis and inflammation can further limit this process (88). The goal of any immunotherapy is to rectify this imbalance in order to restore hepatocytes proliferation and liver functioning (89, 90).

## Macrophages therapy

Recent studies have showed that macrophages affect hepatic fibrous tissue accumulation and decrease. Thus, several are investigating macrophages therapy to cure liver fibrosis, eliminate inflammation, and regenerate the liver (91, 92). A study found that alternative activated macrophages (AAMs) exhibit a high level of phagocytic activity. When AAMs were injected intravenously into mice, it resulted in a reduction in hepatic necrotic area, translocation of high-mobility group box 1 (HMGB1), infiltration of neutrophils, and pro-inflammatory cytokines in cases of ALI caused by APAP. Additionally, AAMs also stimulated the proliferation of hepatocytes. Furthermore, the introduction of clinical-grade human AAMs (hAAMs) resulted in a partial replication of the effectiveness observed in immunocompetent mice (93). Furthermore, a completed phase 1 dose-escalation clinical trial of first-in-human autologous macrophages therapy in adult cirrhotic patients with model for end-stage liver disease (MELD) scores of 10-16 (ISRCTN 10368050)^I^, utilized descriptive analysis, with the primary endpoints of safety and feasibility after macrophages infusion, with no serious adverse events, demonstrated that macrophages administration is safe and effective (94).

## Cytokines therapy

**Figure 5:**
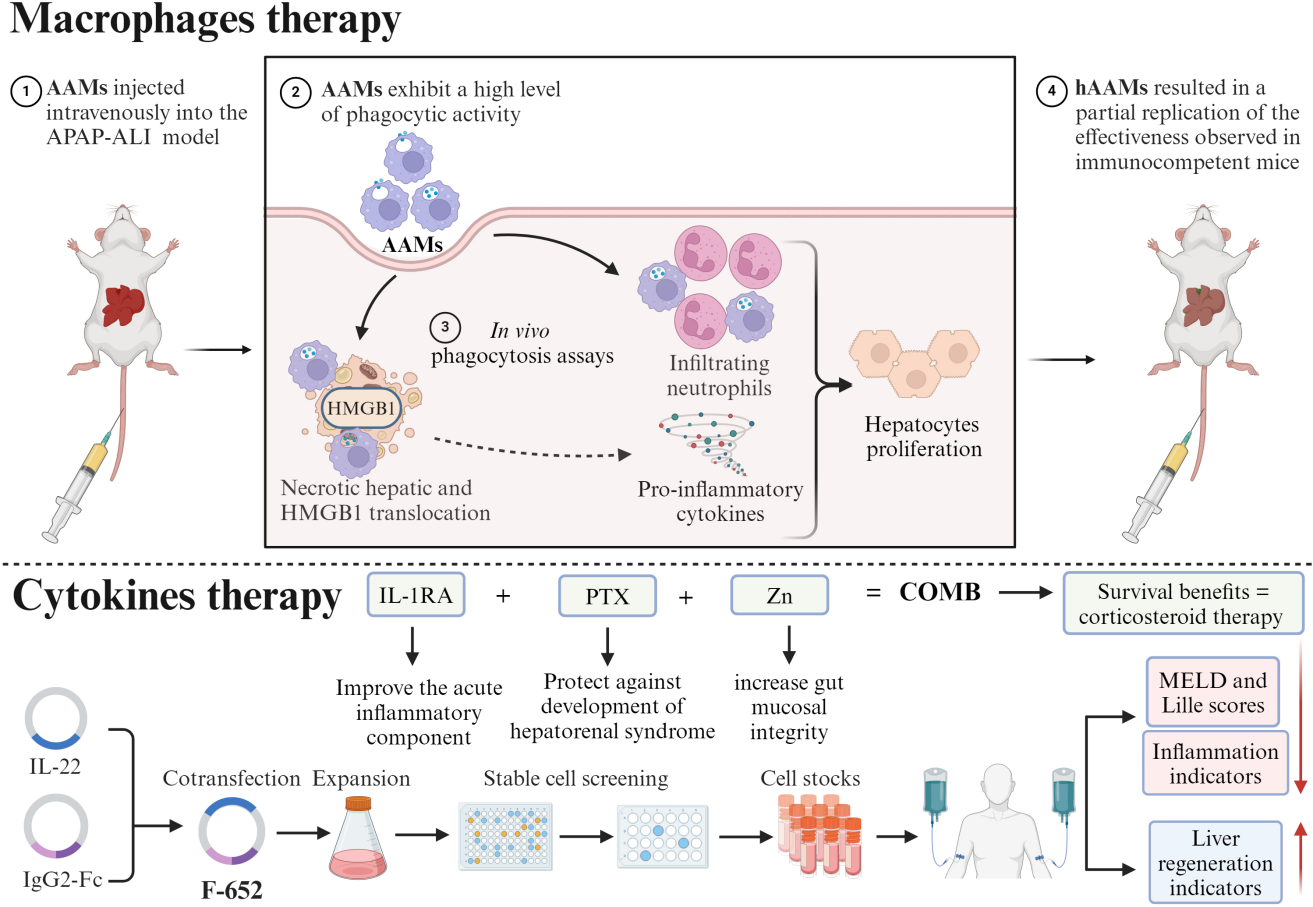
Therapeutic strategies for liver fibrosis. Macrophage-based therapy: In mouse models of APAP-induced ALI, intravenously injected AAMs demonstrate high phagocytic activity. This treatment significantly reduces hepatic necrotic areas, inhibits the translocation of HMGB1, and suppresses neutrophil infiltration and pro-inflammatory cytokine release. Both murine and clinical-grade hAAMs have been shown to stimulate hepatocyte proliferation. Cytokine-based therapy: F-652: A Phase 2a trial in patients with AH demonstrated that F-652 is safe and well-tolerated. Treatment led to significant improvements in Lille and MELD scores, characterized by a decrease in inflammatory markers and an increase in liver regeneration indicators. COMB: A randomized trial utilizing a combination of IL-1RA, Pentoxifylline, and Zn showed a survival benefit comparable to corticosteroids in patients with severe AH, without serious adverse effects. This figure was created using BioRender (https://biorender.com/).

Cytokines produced by immune cells possess antioxidant, anti-apoptotic, anti-steatosis, and antibacterial properties. As a result, they safeguard liver tissue from damage, inflammation, and bacterial infection, while also stimulating the growth of hepatocytes (95, 96). A study found that a completed phase 2a open-label, dose-escalation clinical trial in adult patients with moderate-to-severe AH with MELD scores of 11-28 (NCT 02655510)^II^, treated with a recombinant fusion protein of human IL-22 and IgG2-Fc (F-652) using propensity score matching from independent patient cohorts, with the primary endpoints of safety and tolerability of F-652 and without serious adverse events, demonstrated that F-652 at doses of up to 45μg/kg was safe and was associated with a high rate of improvement as determined by the Lille and MELD scores, decreases in indicators of inflammation and rises in indicators of liver regeneration (97). Moreover, a completed multicenter, randomized, double-blind trial in patients with a clinical diagnosis of severe AH (NCT 01809132)^III^, treated with the combination of anakinra (IL-1RA), pentoxifylline (PTX) plus zinc (Zn) (COMB) in a permuted block design, with a primary endpoint of 180-day mortality and no serious adverse events, demonstrated that COMB offer a similar survival benefit as corticosteroids in the treatment of severe AH (**Figure 5****) (**98).

Recent immunotherapies have shown initial safety and effectiveness in macrophage transplantation and the use of recombinant cytokines, including IL-22 (94). Nevertheless, owing to the liver’s immune microenvironment’s dynamic characteristics and interindividual variability, existing clinical trials are still in preliminary phases and predominantly concentrate on salvage therapy for end-stage liver disease (99). Moreover, addressing the limited lifespan of immune cells *in vivo*, their inadequate targeting efficacy, and the risk of systemic immunological adverse effects is a significant obstacle to extensive clinical use (100). Future immunotherapy should progress towards personalization and combination therapies. Integrating gene-editing technologies, such as CAR-M/T, can improve the precision of cell treatments, while investigating drug delivery systems utilizing biomaterials can facilitate localized, sustained release of cytokines in regions of liver injury (101). The primary objective is to develop a precise intervention program tailored to the patient’s immunological profile, effectively managing inflammation while enhancing the liver’s intrinsic regenerating capacity.

## Conclusion

The advancement of liver disease is a dynamic cycle of damage-inflammation-regeneration, wherein immune regulatory networks function as the fundamental mechanism dictating the illness’s trajectory (99). Recent research has advanced from broad definitions of inflammation to detailed characterizations of cellular phenotypic changes and molecular signaling cascades (102). Immunotherapy is at a pivotal point, moving from experimental investigation to exact clinical use. Future advancements will hinge on three key dimensions: First, employing single-cell spatio-omics and organoid technologies to validate immune regulatory axes identified in animal models with human samples (83); second, transitioning the therapeutic focus from mere anti-inflammation to reprogramming the immune microenvironment, directing immune cells to shift from a pro-fibrotic phenotype to a pro-repair phenotype (103); finally, by amalgamating gene editing technologies with sophisticated biomaterial delivery systems, we can realize highly personalized immune intervention strategies with spatiotemporal precision (104). A comprehensive understanding of the immune regulatory processes involved in liver injury will establish the theoretical basis for the development of highly effective and safe targeted pharmaceuticals and cell treatments, ultimately facilitating the therapeutic objectives of liver functional remodeling and regeneration.
